# Benznidazole Biotransformation and Multiple Targets in *Trypanosoma cruzi* Revealed by Metabolomics

**DOI:** 10.1371/journal.pntd.0002844

**Published:** 2014-05-22

**Authors:** Andrea Trochine, Darren J. Creek, Paula Faral-Tello, Michael P. Barrett, Carlos Robello

**Affiliations:** 1 Unidad de Biología Molecular, Institut Pasteur de Montevideo, Montevideo, Uruguay; 2 The Wellcome Trust Centre for Molecular Parasitology, Institute for Infection, Immunity and Inflammation and Glasgow Polyomics, College of Medical, Veterinary and Life Sciences, University of Glasgow, Glasgow, United Kingdom; 3 Department of Biochemistry and Molecular Biology, Bio21 Molecular Science and Biotechnology Institute, University of Melbourne, Parkville, Victoria, Australia; 4 Departamento de Bioquímica, Facultad de Medicina, Universidad de la República, Montevideo, Uruguay; Northeastern University, United States of America

## Abstract

**Background:**

The first line treatment for Chagas disease, a neglected tropical disease caused by the protozoan parasite *Trypanosoma cruzi*, involves administration of benznidazole (Bzn). Bzn is a 2-nitroimidazole pro-drug which requires nitroreduction to become active, although its mode of action is not fully understood. In the present work we used a non-targeted MS-based metabolomics approach to study the metabolic response of *T. cruzi* to Bzn.

**Methodology/Principal findings:**

Parasites treated with Bzn were minimally altered compared to untreated trypanosomes, although the redox active thiols trypanothione, homotrypanothione and cysteine were significantly diminished in abundance post-treatment. In addition, multiple Bzn-derived metabolites were detected after treatment. These metabolites included reduction products, fragments and covalent adducts of reduced Bzn linked to each of the major low molecular weight thiols: trypanothione, glutathione, γ-glutamylcysteine, glutathionylspermidine, cysteine and ovothiol A. Bzn products known to be generated *in vitro* by the unusual trypanosomal nitroreductase, TcNTRI, were found within the parasites, but low molecular weight adducts of glyoxal, a proposed toxic end-product of NTRI Bzn metabolism, were not detected.

**Conclusions/significance:**

Our data is indicative of a major role of the thiol binding capacity of Bzn reduction products in the mechanism of Bzn toxicity against *T. cruzi*.

## Author Summary

The unicellular parasite *Trypanosoma cruzi* infects humans, leading to Chagas disease, endemic in Central and South America and responsible for 13,000 annual deaths. Only two drugs have proven effective against Chagas, nifurtimox and benznidazole (Bzn). Bzn has the best safety and efficacy profiles and is thus used as first line treatment. Bzn is a pro-drug, and possesses a nitro group which needs to be enzymatically reduced within the parasite to become active. We have investigated for the first time, by means of mass spectrometry based metabolomics, the global changes to small metabolites that occur once Bzn enters the parasite. A decrease in the levels of several thiols, including cysteine and trypanothione, and an increase in gamma-glutamyl containing dipeptides were observed after treatment. Reduced metabolites of Bzn were also detected, together with numerous covalent conjugates of the drug combined with low molecular weight thiols and some non-thiol metabolites. Overall, Bzn treatment primarily affects thiol containing molecules in *T. cruzi*, and this interference with thiol metabolism contributes to the drug's mode of action.

## Introduction

Ten million people worldwide are infected with *Trypanosoma cruzi*, the causative agent of Chagas disease, and 40 million are at risk of infection [1], [2]. In spite of a substantial reduction in prevalence over the last few decades, the disease is considered among the world's 17 most neglected tropical diseases and is responsible for 13,000 annual deaths according to the World Health Organization (WHO). *T. cruzi* is naturally transmitted to humans and other mammals by reduviid insects of the subfamily Triatominae, and may also be transmitted by blood transfusions, organ transplants, orally through contaminated food, and vertically from mother to child. The disease progresses with an initial acute phase, usually asymptomatic, that can subsequently develop into a chronic form with cardiac and digestive pathologies that can lead to death [3].

Benznidazole (Bzn), formerly commercialized as Rochagan and Radanil (Roche), and nifurtimox (Nfx), marketed as Lampit (Bayer), are the only drugs proven effective against Chagas disease. Both contain a nitro group linked, respectively, to an imidazole or furan ring, and unwanted side effects are common, leading to treatment discontinuation in some cases. Bzn has the best safety and efficacy profile, and is therefore used as first line treatment. A major limitation is the low potency of these drugs against parasites in the established chronic disease, which is the form most commonly encountered clinically [4], [5]. The therapeutic benefit of Bzn in established mild to moderate Chagas disease is currently under scrutiny in the Benznidazole Evaluation for Interrupting Trypanosomiasis (BENEFIT) trial [6]. In spite of the limitations of Bzn and Nfx in treatment, only a few compounds are undergoing clinical trials against chronic Chagas disease, and there are no immediate prospects of a vaccine. Interest in nitro-heterocyclic compounds has recently been reinvigorated given the advancement of several members of the class into clinical trials [7], [8], [9].

Bzn was discovered as an anti-trypanosomal agent through screening against parasites without understanding its mechanism of action. Other nitroimidazoles, including the 5-nitroimidazole metronidazole, are well-established in the treatment of anaerobic protozoal and bacterial infections [10]. The mode of action of nitroheterocyclic compounds appears to involve metabolic activation of the compounds initiated through reduction of the compounds' nitro group. Subsequent metabolism of the compounds can be divergent. The anti-mycobacterial agent PA-824, for example, is reduced by a deazaflavin (F_420_)-dependent nitroreductase (Ddn) in *M. tuberculosis* and eventually decomposes to various reactive nitrogen species including nitric oxide [11]. Bzn activity has been proposed to be mediated via reduced intermediates that covalently modify macromolecules *in vivo*, including lipids, DNA and proteins, rather than by formation of radical intermediates producing reactive oxygen species [12], [13]. An unusual prokaryotic type I nitroreductase (NTRI) was identified in trypanosomatid protozoa which is primarily responsible for the reductive activation of some trypanocidal nitroheterocycles including Bzn, Nfx and also fexinidazole [14], [15], [16]. *In vitro* TcNTR can catalyse the consecutive two electron reduction of Bzn, leading to formation of a dihydroxy-dihydroimidazole derivative which may decompose to give glyoxal, a well-known toxic metabolite. Glyoxal was postulated to contribute to the pleiotropic effects of Bzn on trypanosomes [17], although roles for each of the metabolic products of Bzn have not been investigated *in situ*. Here we report, for the first time, an untargeted metabolomics analysis of *T. cruzi* to investigate changes in the parasite associated with exposure to the drug. Using a platform involving HILIC chromatography to separate low molecular weight metabolites coupled to high resolution mass spectrometry [18] we detected in the order of a thousand *T. cruzi* metabolites and were able to identify metabolic perturbations associated with Bzn exposure, as well as the production of Bzn metabolites and their *in situ* reaction products.

## Methods

### Parasite growth and metabolite extraction

*T. cruzi* epimastigotes of the DM28c strain [19] were grown in LIT medium supplemented with yeast extract and 10% foetal bovine serum at 28°C [20]. For metabolite extraction a protocol was adapted from that used for other trypanosomatid protozoa [18], [21]. Cultures were initiated by inoculating exponentially growing epimastigotes to a final concentration of 2.5×10^7^ parasites per mL. Two days after inoculation, parasites were counted in a Neubauer chamber and 1×10^8^ parasites per sample were taken for treatment (approximately 1 mL). After adding 20 or 50 µM Bzn, parasites were incubated at 28°C for 6 h. Tubes containing parasites in suspension were quenched on ice for 3 min, after which Bzn was added to control cells when necessary (Table S1). Cells were collected immediately by centrifugation (2000× g, 4°C, 3 min). Supernatants were carefully removed from cell pellets. Samples from each supernatant were separated for analysis as medium samples (5 µL). Cell disruption and metabolite extraction was performed using 200 µl chloroform/methanol/water 20/60/20 (v/v/v) during 1 hour in a Thermomixer (1000 rpm, 4°C –Eppendorf AG, Hamburg, Germany). Metabolite extracts were separated from cell debris by centrifugation (13,000× g, 4°C, 3 min). Extracts were stored at −70°C under nitrogen gas until analysis. Biological replicates were grown, incubated and extracted on different days. A short drug incubation period was chosen to impede changes in the number of parasites during treatment. For viability determinations, treated parasites were washed, incubated in fresh medium for 72 h and counted in Neubauer chamber.

### Mass spectrometry

Samples were analysed on an Exactive Orbitrap mass spectrometer (Thermo Fisher Scientific) in both positive and negative modes (rapid switching), coupled to a HPLC separation with a ZIC-HILIC column (Sequant) as has previously been described [18]. All samples from each experiment were analysed in the same analytical batch in randomised order and the quality of chromatography and signal reproducibility were checked by analysis of quality control samples, internal standards and total ion chromatograms. A standard mix containing approximately 160 authentic metabolite standards was run at the start of each analysis batch to aid metabolite identification.

MSMS analysis was performed on an LTQ Orbitrap Velos mass spectrometer (Thermo Fisher Scientific) in positive mode with ZIC-HILIC chromatography as described above. High resolution (15,000) MSMS spectra were obtained with HCD induced fragmentation at normalised collision energy of 35 eV. MSMS spectra for the follow-up 50 µM Bzn experiment were collected at unit resolution.

### Data processing

Untargeted metabolite analysis was conducted with the freely available software packages mzMatch [22] and Ideom (http://mzmatch.sourceforge.net/ideom.php) [23]. Raw LC-MS data was converted to mzXML format and peak detection was performed with XCMS [24] and saved in peakML format. Mzmatch.R was used for sample alignment, peak filtering (based on reproducibility, peak shape and an intensity threshold of 3000), gap filling and annotation of related peaks. Ideom was used to remove contaminants and LC-MS artefact peaks and to perform metabolite identification. Metabolite identities were confirmed by exact mass (within 3 ppm after correction for loss or gain of a proton in negative mode or positive mode ESI respectively) and retention time where authentic standards were available for analysis. Putative identification of all other metabolites was made on the basis of exact mass and predicted retention time from all metabolites from the KEGG, MetaCyc and Lipidmaps databases [18]. The m/z values corrected for proton gain or loss are referred as m/z_c_ in the text. In cases where identification was putative, the most likely metabolite was chosen based on available chemical and biological knowledge [23]. However, LC-MS data alone is often insufficient for accurate isomer identification and lists of alternative identifications with meta-data for each identified formula are accessible in the macro-enabled Ideom files (Files S1 and S2; help documentation available at mzmatch.sourceforge.net/ideom.php). Quantification is based on raw peak heights, and expressed relative to the average peak height observed in untreated cells from the same experiment. Unidentified peaks in the LC-MS data were also investigated for drug-induced changes. After removal of LC-MS artefacts and known contaminants, measured exact masses were compared with theoretical exact masses of Bzn derived metabolites contained in Bznmet database (“targeted sheet”, Files S1 and S2). This database included reported and putative Bzn reduction products and covalent adducts of Bzn with small cellular metabolites, all retrieved from existing reports on *in vivo* and *in vitro* modification processes for Bzn and similar nitroimidazoles, covering enzymatic and non-enzymatic conversions [17], [25], [26], [27]. Accurate mass and relative isotope abundance was used to determine the chemical formulae for the remaining unidentified metabolites detected specifically in the 50 µM Bzn treated samples. In most cases these formulae contain the subset C_12_H_14_N_4_O, suggesting that they are adducts of reduced Bzn with a broad range of unexpected metabolites. In addition, a targeted analysis of potential metabolites in the Bznmet database was performed on the raw data using accurate mass within a 3 ppm mass range, to allow detection of metabolites that may have been excluded by the automated data processing due to peak shape, intensity or reproducibility filters. Manually retrieved intensity and RT values from all samples are included in File S3.

### Accession numbers

TcNTRI: GenBank AHD24669.1

TbNTRI: GenBank AAX69576.1

Dnd *Mycobacterium tuberculosis*: UniProtKB/Swiss-Prot P71854.1

TcCPR-B: GenBank ABI15738.1

TcOYE: GenBank AAX54861.1

TcAKR: GenBank ACD93222.1

## Results

### General description of the *Trypanosoma cruzi* metabolome

Metabolites were extracted from *T. cruzi* epimastigote cell pellets with a monophasic solvent mixture of chloroform, methanol and water (1∶3∶1); separated and analysed by ZIC-HILIC chromatography coupled to high accuracy MS using an Orbitrap mass spectrometer. Five biological replicates were analysed for each condition. Signal extraction and initial filtering of the LC-MS data yielded 3,117 peaks for negative mode and 5,528 peaks for positive ESI mode. Additional artefact filtering and polarity merging in Ideom reduced the list of features to 1,477 candidate molecules, of which over 70% (1,069) matched compounds in the metabolite databases based on accurate mass and retention time information [18]. A complete list of putatively identified metabolites, with the detected peak heights for each sample and confidence values for their identifications is supplied in File S1 (see “Comparison” sheet). Only putatively identified metabolites were considered for intensity comparisons among groups of samples.

**Figure 1:**
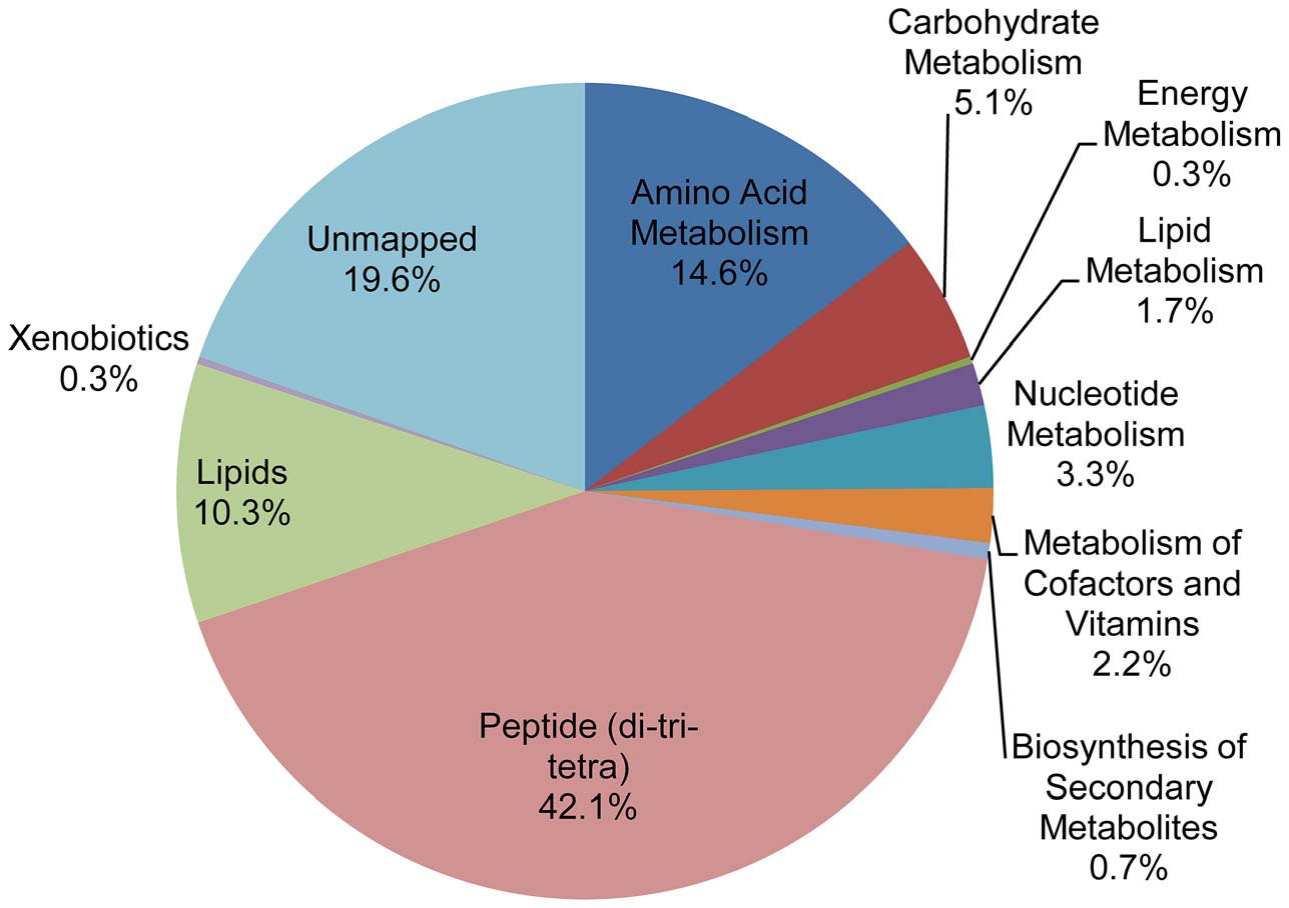
Distribution of total metabolites identified in *Trypanosoma cruzi* epimastigote samples. The pie chart depicts the percentages of putatively identified metabolites from each of the metabolite classes. A total of 1,069 metabolites were analysed.

Based on the Ideom software's automated metabolite calling we can divide metabolites into several classes, although additional confirmation of the identity of each individual metabolite would be required to impose certainty on these classifications. The largest class of metabolites was peptides, representing 42.1% of the total metabolites (135 dipeptides, 183 tripeptides and 132 tetrapeptides) (Figure 1 and File S1). The next largest class of metabolites was amino acids and compounds associated with amino acid metabolism (amino acids, thiol compounds and polyamines), which represented 14.6% of the total putatively identified metabolites.

**Figure 2:**
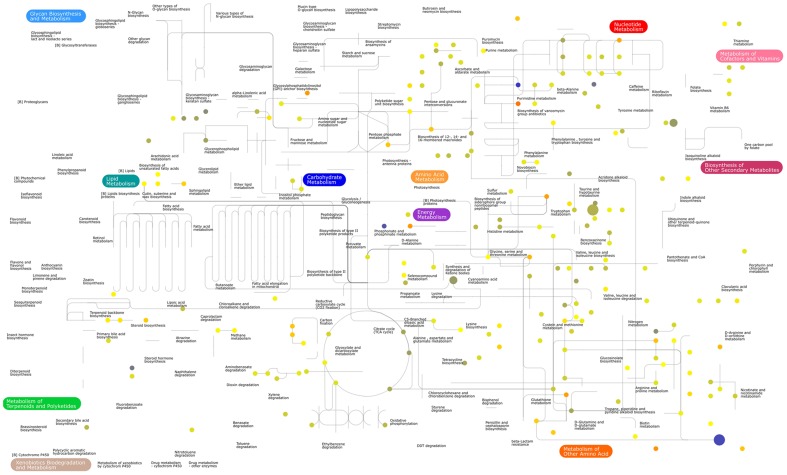
Putatively identified metabolites mapped onto the KEGG metabolic pathways. Coloured nodes (putatively identified metabolites) demonstrate coverage of diverse metabolic pathways (grey lines indicate annotated *T. cruzi* pathways). Node colour represents metabolite abundance in Bzn-treated *T. cruz*i relative to untreated controls on a continuous colour scale: blue: 0.5, yellow: 1 (no change), red: 2-fold increase. Node size indicates P-value from unpaired Welch's t-test: large: p<0.01, medium: p<0.05, small: P≥0.05. The large blue spot in the lower-right corner represents trypanothione disulphide (relative abundance = 0.7, P-value<0.01). Image generated with iPath2.0 [66].

In addition to peptides and amino acids, metabolites from a diverse range of metabolic pathways were detected (Figure 2), including lipid (110), carbohydrate (44), nucleotide (33) and cofactor metabolism (26). Putatively identified metabolites that lack KEGG [28] or Lipidmaps [29] pathway annotations were classified as unmapped (210), among these there are N-acetylated amino acids and polyamines, acyl-carnitines and acyl-glycines.

A hallmark metabolite in kinetoplastid parasites, including trypanosomes, is trypanothione. This di-thiol is composed of two molecules of glutathione linked by one molecule of spermidine, and it is usually detected as a multi charged ion by ESI-MS [30]. Under the conditions used in this study, the tri-charged form of trypanothione disulphide was detected in all parasite samples with very high S/N ratios. Furthermore, a mass consistent with tri-charged homotrypanothione, a metabolite unique to *T. cruzi* [31], was also detected although with eighty times lower signal intensity than trypanothione. It is important to note that the protocol used here leads to extensive oxidation of thiols, thus the relative quantification of the intracellular redox state of these molecules is not possible. Other common small thiols detected were glutathione, cysteine/cystine and homocysteine. Detected polyamines included cadaverine, spermidine, putrescine and some modified forms such as N-acetylspermidine, N-acetylputrescine and gamma-glutamylputrescine.

### Metabolic profile of benznidazole treated *Trypanosoma cruzi*

In an attempt to analyse the metabolic changes induced by Bzn treatment, *T. cruzi* epimastigotes were exposed to 20 µM Bzn over six hours (cBt samples) after which metabolites were extracted. Approximately 80% of the parasites remained viable after this treatment (not shown). Parasites that were not exposed to Bzn (cTc) and parasites to which Bzn was added just prior to the extraction of metabolites (cBc) (to control for any mass spectrometry related effects due to drug) were included as controls. Medium samples were analysed in parallel including fresh medium (Med) and spent mediums collected from cBc (mBc) and cBt samples (mBt).

**Figure 3:**
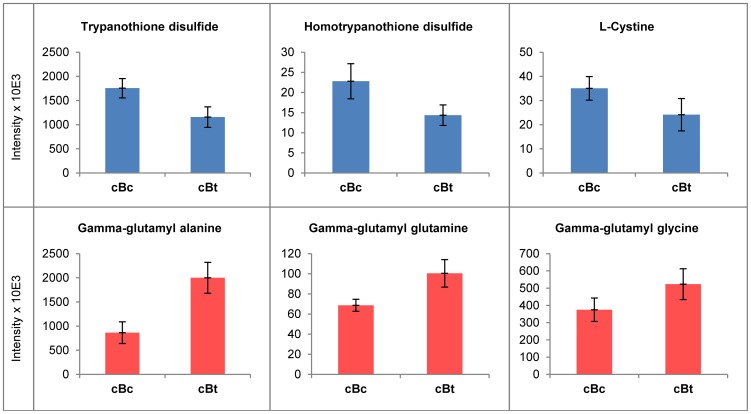
Metabolites displaying significant differences between control and 20 µM Bzn treated *T. cruzi* samples. Metabolites displaying significant differences between control samples (cBc) and 20 µM Bzn treated samples (cBt) with p<0.05 (unpaired T-test) and fold change >1.4. Bzn derived metabolites were not included.

Principal Component Analysis (PCA) of the automatically filtered data indicated that no obvious differences were found among the different groups of cells samples (cBc, cBt and cTc), although medium samples could be readily separated from cell samples (Figure S1). This indicated that the global structure of the *T. cruzi* metabolome was little changed by this treatment with Bzn. Univariate analysis of individual metabolites using p<0.05 as a significance threshold in t-tests and a fold abundance change above 1.4 revealed relatively few differences between treated and untreated parasites (see “Comparison” sheet, File S1). Among these, trypanothione disulphide, homotrypanothione disulphide and cystine (cysteine disulphide) were significantly diminished in abundance after treatment. Three glutamate containing di-peptides were elevated in Bzn treated samples (Figure 3), and although they were not structurally characterised, they likely represent the gamma-glutamyl dipeptides involved in glutathione recycling.

In addition to the relatively small changes in the metabolites discussed above, two metabolites which were substantially elevated in treated cells over controls were identified through the automated screening of the datasets. One metabolite (m/z_c_ 278.14, RT 11.1) was assigned as N-Benzoyl-D-arginine and another (m/z_c_ 292.15, RT 10.2) as 4-coumaroyl-3-hydroxyagmatine, based on mass similarity. However, as discussed below, these were mis-identifications of Bzn metabolites that have masses (i.e. molecular formulae) identical to these representatives from the IDEOM metabolite database.

Considering that Bzn acts as a pro-drug which undergoes reductive metabolism to generate toxic intermediates [4], [5], [13], the presence of unidentified peaks at significantly higher concentrations in treated than in untreated cells prompted us to re-scan the filtered data seeking putative molecules derived from *in situ* Bzn metabolism. We applied a correlation analysis on the intensities of all the LC-MS peaks, searching for molecules that were highly correlated with the metabolites detected in treated samples (and thus assumed to be derived from Bzn) and with low or no abundance in all the other samples (control parasites, media and solvent samples) (“all Data” sheet, File S1). Six additional ions corresponding to putative Bzn metabolites were found after carefully removing additional MS artefacts derived from these molecules (Table 1).

**Table 1:** Bzn *in vivo* derived metabolites arising after 20 µM Bzn treatment of *T. cruzi* epimastigotes.

RT (min)	m/z_c_	IS	Relative isotope abundance	Proposed formula	Mass error (ppm)	Mean cBt (×10^3^)	Proposed metabolite	N°
**10.9**	230.1169	1	^13^C: 13%	C_12_H_14_N_4_O	0.4	2150	Amino der.	**1**
**11.1**	278.1378	1	^13^C: 11%	C_13_H_18_N_4_O_3_	−0.2	430	Amino methoxy der.	**2**
**13.0**	264.1222	1	^13^C: 10%	C_12_H_16_N_4_O_3_	−0.3	145	Amino dihydroxy dihydro der.	**3**
**18.5**	276.5977	2	^13^C: 22% ^34^S: 3%	C_22_H_31_N_7_O_8_S	−0.3	36	Amino der.+glutathione+H_2_O	**4**
	553.1960	1	^13^C: 22% ^34^S: nd		0.9	16		
**11.7**	206.1167	1	^13^C: 5%	C_10_H_14_N_4_O	−0.3	33	N-benzyl-2-guanidinoacetamide	**5**
**10.2**	292.1536	1	^13^C: 12%	C_14_H_20_N_4_O_3_	0.1	30	Amino dimethoxy der.	**6**
**17.6**	267.5922	2	^13^C: 20% ^34^S:3%	C_22_H_29_N_7_O_7_S	−1.1	20	Amino der.+glutathione (isomer 1)	**7**
	535.1848	1	^13^C: 20% ^34^S: nd		−0.2	5.3		
**18.4**	267.5923	2	^13^C: 19% ^34^S:1%	C_22_H_29_N_7_O_7_S	−0.6	9.9	Amino der.+glutathione (isomer 2)	**8**
	535.1858	1	^13^C: 17% ^34^S: 1%		1.6	7.2		
**11.0**	349.1208	1	^13^C: 12% ^34^S: nd	C_15_H_19_N_5_O_3_S	−0.3	9.5	Amino der.+cysteine	**9**
**29.0**	322.4667	3	^13^C: 47% ^34^S: nd*	C_39_H_61_N_13_O_12_S_2_	−0.2	7.7	Amino der.+trypanothione-SH+O	**10**
**11.0**	246.1116	1	^13^C: 9%	C_12_H_14_N_4_O_2_	−0.4	5.0	hydroxylamine der. or hydroxy der. 1 or 2	**11**
**21.5**	214.5789	2	^13^C: 15% ^34^S: 2%	C_19_H_23_N_7_O_3_S	−1.1	3.7	Amino der.+ovothiol A (isomer 2)	**12**
	429.1584	1	nd		0.2	5.6		
**28.0**	316.4631	3	^13^C: 41% ^34^S: nd*	C_39_H_59_N_13_O_11_S_2_	−0.7	2.0	Amino der.+trypanothione double bonded	**13**
**19.5**	214.5791	2	nd	C_19_H_23_N_7_O_3_S	−0.2	0.6	Amino der.+ovothiol A (isomer 1)	**14**
	429.1577	1	nd	C_19_H_23_N_7_O_3_S	−1.3	0.1		
**34.5**	220.7773	3	nd	C_29_H_46_N_10_O_6_S	−0.4	0.5	Amino der.+glutathionylspermidine	**15**
**29.9**	356.8040	3	^13^C: 34% ^34^S: nd*	C_42_H_66_N_14_O_13_S_3_	2.3	0.4	Amino der.+trypanothione-S-S-Cys	**16**

Ions detected from 20 µM Bzn treated parasites (cBt samples) and with low or no abundance in control samples (cBc, cTc, medium and solvent samples) are listed, retrieved from filtered or raw data. **RT**: retention time. **m/z_c_:** m/z values corrected for proton gain or loss (m/z_c_ = observed *m/z* ± 1.007276). **IS**: Ionization state. 1, 2 and 3 refer to mono, di and tri charged ions respectively, from positive or negative ESI modes. Ionization was manually confirmed for all the listed metabolites, examining the m/z values of the ^13^C related isotopic peaks. **Relative isotope abundance**: values from isotopic peaks were retrieved from filtered data or raw data. **Proposed formula**: most formulae were retrieved from BznMet database proposed metabolites. Some formulae were predicted using m/z data with IDEOM and rCDK [67]. **Mass error (ppm):** [(m/z(observed)-m/z(exact))/m/z(exact)]*1E+6. **Mean cBt**: the mean peak intensity value for each ion in the corresponding study group (cBt). **Proposed metabolites**: proposed metabolites for each ion are listed with complete names or with short assigned names which include some selected features of the metabolites. der.: derivative. Complete IUPAC names and SMILES codes are included on File S1 “Targeted sheet”. All intensity values from raw data were retrieved manually (File S3). **Metabolite number:** numbers were assigned for cross referencing.

*^34^S isotopic peaks were not resolved from the ^13^CII peaks in these metabolites.

To further investigate the identity of the Bzn metabolites and to search for additional Bzn metabolites, an in-house database of reported and putative Bzn metabolites was built: Bznmet database (“Targeted” sheet, File S1). The theoretical m/z_c_ values contained in the database (109 ions belonging to 56 different molecules) were compared with the accurate measured m/z_c_ values from the collected filtered data and also the raw data, resulting in identification of a number of Bzn metabolites (Table 1 and File S3). Finally, to gain additional information to support the proposed metabolite structures, MSMS fragmentation spectra were collected for the ions of interest (File S4).

Once the LC-MS data were examined, a number of reduced derivatives of Bzn were found to be highly abundant in treated samples. The signal at m/z_c_ 264.12 (RT 13 min) was attributed to a dihydroxy-dihydro derivative of Bzn: 2-(2-amino-4,5-dihydroxy-4,5-dihydroimidazol-1-yl)-N-benzylacetamide (**3**). Additionally, the two metabolites initially detected in Bzn-treated samples and mis-identified, as N-Benzoyl-D-arginine (m/z_c_ 278.13, RT 11.1 min) and 4-coumaroyl-3-hydroxyagmatine (m/z_c_ 292.15, RT 10.2 min), could be assigned as methoxy derivatives of the Bzn dihydroxy-dihydro derivative (**2**, **6**). Also, a molecule with an m/z value expected for the hydroxylamine or some hydroxy derivatives of Bzn was found (m/z_c_ 246.11, RT 11 min) (**11**). N-benzyl-2-guanidinoacetamide (m/z_c_ 206.11, RT 11.7 min) (**5**) was also detected. This molecule was reported to arise after the Bzn dihydroxy-dihydro derivative decomposes, or reacts with other molecules, to release glyoxal [17], [32]. Nevertheless, glyoxal adducts with nucleotides, nitrogenated bases or amino acids could not be detected in our data. Also, 2-(2-amino-1H-imidazol-1-yl)-N-benzylacetamide (m/z_c_ 230.11, RT 10.85 min) (**1**), a six electron reduction product of Bzn, was highly abundant in treated samples.

Multiple detected signals were assigned to covalent adducts of Bzn reduction products with low molecular weight thiols. Three signals were assigned to different Bzn-trypanothione adducts, all detected as tri-charged ions (m/z_c_ 322.47, RT 29.1; m/z_c_ 316.46, RT 28 and m/z_c_ 356.8, RT 29.9 min) (**10**, **13**, **16**). Likewise, a glutathionylspermidine adduct was detected as a tri-charged ion (m/z_c_ 220.78, RT 34.5 min) (**15**). The mono-charged ion with m/z_c_ 349.12 (RT 11 min) was assigned to a cysteine-containing adduct (**9**). In addition, we found a glutathione adduct with reduced Bzn, both as a mono and a di-charged ion, with m/z_c_ 535.18 and 267.59 respectively. Each of these ions were detected at two different retention times (RT 17.6 and 18.4 min), most probably representing two isomers (**7**, **8**). Ovothiol A covalent adducts were assigned to signals present at two retention times on HILIC chromatography (m/z_c_ 429.16 and m/z_c_ 214.58; both at RT 19.5 and 21.5 min) (**14**, **12**).

High quality MSMS spectra were obtained for a number of putative Bzn-derived molecules, even though some metabolites were detected with very low intensity signals. The analysis of the fragmentation patterns is summarized in File S4. Although additional structural data would be necessary to unequivocally identify all the metabolites, the fragmentation data, together with the high resolution accurate mass MS and RT data, are supportive for the proposed structures. In this sense, all MSMS spectra contained a fragment of m/z_c_ 90.047 assigned to the benzene ring moiety of Bzn and also present in the Bzn fragmentation pattern. In addition, molecules proposed as related structures displayed fragmentation patterns with shared peaks. Metabolites with m/z_c_ 264.12 (**3**) and 278.13 (**2**) (hydroxy and methoxy derivatives) included 14 shared signals, while cysteine (**9**) and glutathione adducts (**7**, **8**) (Bzn-thiol conjugates) displayed five shared peaks. Furthermore, shared peaks were obtained when glutathione and trypanothione MSMS fragmentation spectra were compared with the corresponding spectra of the proposed Bzn-thiol adducts. Among the common peaks we found the typical fragment encountered in glutathione containing molecules of mass 129 Da [33] and also fragment masses that correspond to the neutral loss of pyroglutamic acid. Also, ^34^S isotopic peaks were observed for a number of metabolites, confirming the presence of thiol moieties in these covalent adducts (Table 1).

Finally, a group of cell-free control samples were analysed separately: a control group in which the drug was incubated with the growth medium was compared with the medium alone (not-shown). In this analysis we observed that no Bzn metabolites arise after the drug incubation with the medium components after 6 hours at 28°C. Thus, all of the observed Bzn derived metabolites are produced by *T. cruzi*-mediated metabolism of the drug.

### Additional Bzn derived metabolites are identified using a higher concentration of drug

Since a number of the Bzn-related metabolites were found only with very low abundance when *T. cruzi* epimastigotes were incubated with 20 µM Bzn over six hours, we collected additional metabolomics data using a higher concentration of Bzn, to allow the detection of additional signals arising from Bzn derived molecules and to observe effects on endogenous metabolites. For this purpose, parasites were treated with 50 µM Bzn over 6 h in the same general conditions (cBzt samples). Parasites that were not exposed to Bzn (cBec) and parasites to which Bzn was added just prior to the extraction of metabolites (cBzc) were used as controls. Fifty percent (50%) of the parasites remained viable after this treatment compared to untreated controls (not shown).

After data filtering, and analogous to the 20 µM Bzn treatment, we observed several endogenous metabolites that showed significant differences between treated and control samples (File S2). Consistent with the lower Bzn dose, trypanothione and homotrypanothione showed diminished levels after drug exposure, probably a consequence of the formation of the drug-thiol conjugates. Three lipids putatively identified as vitamin D-related sterols showed diminished levels after treatment, whereas two long-chain acyl-carnitines showed augmented levels, along with the dipeptide γ-glutamylcysteine (T-test P values<0.05 and FC>2).

Treatment with 50 µM Bzn induced the appearance of a high number of Bzn related signals, with 124 ions included in the filtered data. After careful removal of LC-MS artefact ions, 36 of these Bzn-specific peaks were listed as putative Bzn metabolites (Table 2), although some of the analysed signals may still represent in-source MS-derived fragments or adduct ions from true Bzn metabolites. The raw data was scanned for putative Bzn metabolites contained in the Bznmet database, and 14 additional ions were retrieved (Table 2).

**Table 2:** Bzn *in vivo* derived metabolites arising after 50 µM Bzn treatment of *T. cruzi* epimastigotes.

RT (min)	m/z_c_	IS	Relative isotope abundance	Proposed formula	Mass error (ppm)	Mean cBzt (×10^3^)	Proposed metabolite	N°
**11.6**	230.1168	1	^13^C: 13%	C_12_H_14_N_4_O	0.2	36267	amino der.	**1**
**11.9**	278.1381	1	^13^C: 11%	C_13_H_18_N_4_O_3_	0.8	5257	methoxy der. 1	**2**
**13.5**	264.1220	1	^13^C: 11%	C_12_H_16_N_4_O_3_	−0.9	4200	dihydroxy-dihydro der.	**3**
**18.7**	276.5979	2	^13^C: 23% ^34^S: 3%	C_22_H_31_N_7_O_8_S	0.6	2983	amino der.+glutathione+H_2_O	**4**
	553.1952	1	^13^C: 20% ^34^S: 2%		−0.5	549		
**12.5**	206.1165	1	^13^C: 8%	C_10_H_14_N_4_O	−1.3	480	N-benzyl-2-guanidinoacetamide	**5**
**10.9**	292.1534	1	^13^C: 12%	C_14_H_20_N_4_O_3_	−0.5	992	dimethoxy der.	**6**
**17.8**	267.5926	2	^13^C: 21% ^34^S: 3%	C_22_H_29_N_7_O_7_S	0.5	2592	amino der.+glutathione (isomer 1)	**7**
	535.1852	1	^13^C: 20% ^34^S: 3%		0.5	776		
**18.6**	267.5926	2	^13^C: 23% ^34^S: 3%	C_22_H_29_N_7_O_7_S	0.5	3400	amino der.+glutathione (isomer 2)	**8**
	535.1850	1	^13^C: 20% ^34^S: 3%		0.2	646		
**11.9**	349.1209	1	^13^C: 8% ^34^S: nd	C_15_H_19_N_5_O_3_S	0.1	70	amino der.+cysteine	**9**
**30.0**	322.4665	3	^13^C: 42% ^34^S: nd*	C_39_H_61_N_13_O_12_S_2_	−1.1	23	amino der.+trypanothione-SH+O	**10**
	483.7003	2	^13^C: 36% ^34^S: nd*		0.1	0.2		
**11.9**	246.1116	1	^13^C: 10%	C_12_H_14_N_4_O_2_	−0.3	235	hydroxylamine der. or hydroxy der. 1 or 2	**11**
**22.3**	214.5792	2	^13^C: 15% ^34^S: 3%	C_19_H_23_N_7_O_3_S	0.2	507	amino der.+ovothiol A (isomer 2)	**12**
	429.1581	1	^13^C: 16% ^34^S: 3%		−0.5	106		
**29.3**	316.4633	3	^13^C: 44% ^34^S: 7%	C_39_H_59_N_13_O_11_S_2_	0.1	37	amino der.+trypanothione C4 & C5	**13**
**19.6**	429.1587	1	^13^C: nd ^34^S: nd	C_19_H_23_N_7_O_3_S	0.9	0.7	amino der.+ovothiol A (isomer 1)	**14**
	214.5791	2	^13^C: 12% ^34^S: nd		−0.4	0.1		
**35.7**	220.7772	3	^13^C: 28% ^34^S: nd*	C_29_H_46_N_10_O_6_S	−1.1	14	amino der.+glutathionylspermidine	**15**
	331.1659	2	^13^C: 24% ^34^S: nd		−0.6	7.6		
**11.9**	293.1125	1	^13^C: 10%	C_12_H_15_N_5_O_4_	0.3	268	possible nitro adduct of metabolite **3**	**17**
**11.2**	402.6748	2	^13^C: 39% ^34^S: 5%	C_37_H_55_N_7_O_9_S_2_	−0.8	146	possible Bzn-thiol adduct	**18**
**13.5**	246.1113	1	^13^C: 11%	C_12_H_14_N_4_O_2_	−1.4	110	hydroxylamine der. or hydroxy der. 1 or 2	**19**
**17.5**	239.0817	2	^13^C: 19% ^34^S: 2%	C_20_H_26_N_6_O_6_S	−0.1	102	amino der.+γ-glutamylcysteine (isomer 1)	**20**
	478.1636	1	^13^C: 13% ^34^S: nd		0.3	33		
**12.0**	614.3539	1	^13^C: 29%	C_30_H_46_N_8_O_6_	−0.2	80	possible Bzn adduct	**21**
**13.6**	164.0947	1	^13^C: 3%	C_9_H_12_N_2_O	−1.4	67	2-amino-N-benzylacetamide	**22**
**12.6**	248.1273	1	^13^C: 5%	C_12_H_16_N_4_O_2_	−0.1	62	hydroxy der. 3	**23**
**11.9**	398.1800	1	^13^C: 16%	C_17_H_26_N_4_O_7_	−0.4	54	possible Bzn adduct	**24**
**15.9**	275.5901	2	^13^C: 27% ^34^S: 2%	C_22_H_29_N_7_O_8_S	0.7	33	amino der.+glutathione+O	**25**
**11.9**	384.1646	1	^13^C: 15%	C_16_H_24_N_4_O_7_	0.3	31	possible Bzn adduct	**26**
**10.3**	357.1433	1	^13^C: 18%	C_17_H_19_N_5_O_4_	−1.1	29	amino der.+pyroglutamate	**27**
**23.4**	367.1130	2	^13^C: 29%	C_28_H_34_N_10_O_14_	0.5	22	possible Bzn adduct	**28**
**30.3**	314.1182	4	^13^C: 55% ^34^S: nd	C_49_H_76_N_16_O_17_S_3_	−0.7	21	amino der.+trypanothione-S-S-glutathione	**29**
	418.8246	3	^13^C: 59% ^34^S: nd*		0.0	4.6		
**8.6**	276.1225	1	^13^C: 8%	C_13_H_16_N_4_O_3_	0.9	20	methoxy der. 2	**30**
**18.3**	239.0813	2	^13^C: 17% ^34^S: 1%	C_20_H_26_N_6_O_6_S	−1.3	19	amino der.+γ-glutamylcysteine (isomer 2)	**31**
	478.1636	1	^13^C: 13% ^34^S: nd		−0.5	4.2		
**21.8**	407.8620	3	^13^C: 57%	Unknown	nd	14	Unknown MW 1223.59	**32**
**9.9**	377.1154	1	^13^C: 7% ^34^S: nd	C_16_H_19_N_5_O_4_S	−1.0	11	possible Bzn adduct	**33**
**12.7**	434.2067	1	^13^C: 18%	C_23_H_26_N_6_O_3_	0.1	11	possible Bzn adduct	**34**
**23.9**	207.5712	2	^13^C: 16% ^34^S: 1%	C_18_H_21_N_7_O_3_S	−0.6	11	amino der.+mercaptohistidine	**35**
**10.5**	345.1800	1	^13^C: 12%	C_17_H_23_N_5_O_3_	−0.3	9.7	amino der.+valine	**36**
**19.2**	284.0675	1	^13^C: 4% ^34^S: 2%	C_8_H_16_N_2_O_7_S	−1.1	8.4	Unknown MW 284.06	**37**
**10.7**	276.1043	1	^13^C: 6% ^34^S: nd	C_13_H_16_N_4_OS	−0.7	7.3	amino der.+methylthiol	**38**
**12.3**	358.1044	1	^13^C: 13%	C_13_H_19_N_4_O_6_P	0.5	7.2	phosphate ester of methoxy der.	**39**
**10.8**	355.1491	1	^13^C: 12%	C_14_H_21_N_5_O_6_	−0.2	5.8	possible Bzn adduct	**40**
**32.4**	393.1691	3	^13^C: 56% ^34^S: nd	C_51_H_73_N_17_O_12_S_2_	0.5	3.0	amino der.×2+trypanothione	**41**

Ions detected from 50 µM Bzn treated parasites (cBzt samples) and with low or no abundance on control samples (cBzc and cBec samples) are listed retrieved from filtered or raw data. **RT**: retention time. **m/z_c_:** values are corrected for proton gain or loss (m/zc = observed *m/z* ± 1.007276). **IS**: Ionization state. 1, 2, 3 and 4 refer to mono, di, tri and tetra charged ions respectively; from positive or negative ESI modes. Ionization was manually confirmed for all the listed metabolites, examining the m/z values of the ^13^C related isotopic peaks. **Relative isotope abundance**: values from isotopic peaks were retrieved from filtered data or raw data. **Proposed formula**: most formulae were retrieved from BznMet database proposed metabolites. Some formulae were obtained using m/z data with IDEOM and rCDK [67]. **Mass error (ppm):** [(m/z(observed)-m/z(exact))/m/z(exact)]*1E+6. **Mean cBzt**: the mean peak intensity value for each ion in the cBzt group. **Proposed metabolites**: proposed metabolites for each ion are listed with complete names or short assigned names which include some selected features of the metabolites. der.: derivative. Complete IUPAC names and SMILES codes are included on File S2 “Targeted sheet”. All intensity values from raw data were retrieved manually (File S3). **N°:** metabolite numbers were assigned for cross referencing.

*^34^S isotopic peaks were not resolved from the 13CII peaks in these metabolites.

**Figure 4:**
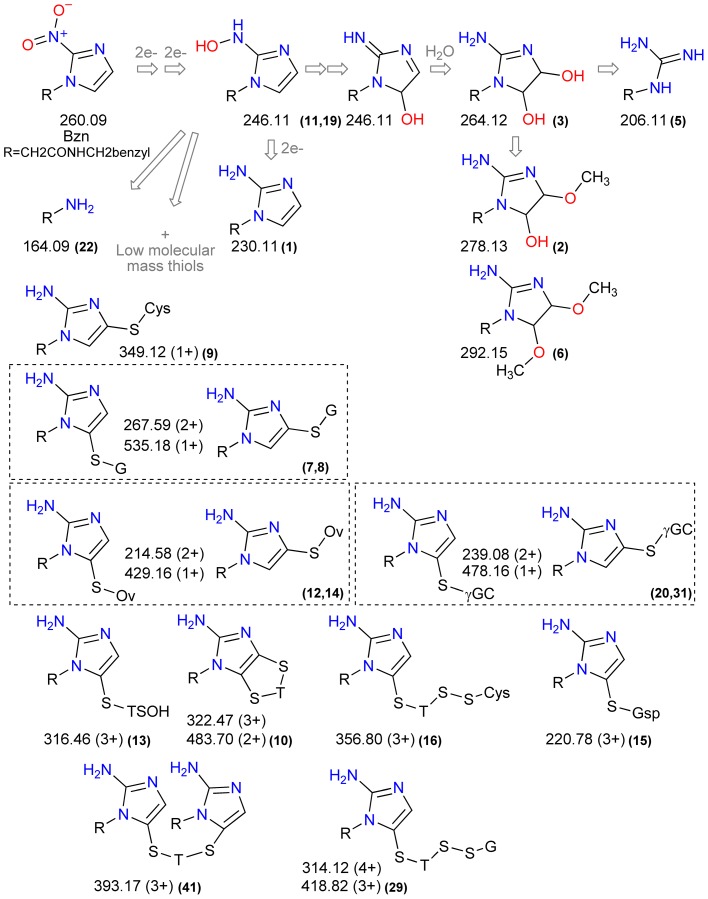
Bzn-derived metabolites. A number of Bzn derived molecules detected after treatment of *T. cruzi* epimastigotes with 20 µM and/or 50 µM Bzn are represented. A putative pathway for their *in vivo* formation is shown; double arrows indicate the existence of possible intermediates such as nitroso or nitrenium derivatives. The observed *m/z* values corrected for proton gain or loss (observed *m/z* ± 1.007276) are included. Cys, G, Ov, γGC, T and Gsp refer to cysteine, glutathione, ovothiol A, gamma-glutamylcysteine, trypanothione and glutathionylspermidine respectively. All thiols are represented with their functional -S- groups separately. Bold numbers in parenthesis are the metabolite reference numbers (Tables 1 and 2).

The Bzn metabolites identified using 20 µM Bzn were also found in the 50 µM assay, with the exception of a metabolite with m/z_c_ 356.80 (**16**). An additional signal at m/z_c_ 164.09 (RT 13.6 min) was found and attributed to 2-amino-N-benzylacetamide (**22**). This compound was described as one of the products of the *in vitro* enzymatic conversion of Bzn by NTRI [17]. The metabolite with m/z_c_ 246.11 was found in both positive and negative ESI modes and with two different retention times (12 and 13.5 min) (**11**, **19**). These could represent both the Bzn hydroxylamine and/or hydroxy metabolites. Also, a conjugate of reduced Bzn with γ-glutamylcysteine was detected in 50 µM Bzn treated samples (m/z_c_ 478.16 and m/z_c_ 239.08, RT 17.5 and 18.3 min) (**20**, **31**). The signal at m/z_c_ 393.17 (RT 32.4 min) was assigned to a molecule composed of trypanothione linked to two reduced Bzn molecules (**41**). A triple and a quadruple charged ion were both attributed to a conjugate of reduced Bzn with a mixed disulphide of trypanothione and glutathione (m/z_c_ 418.82 and m/z_c_ 314.12 at RT 30.3 min) (**29**). The remaining adducts included mercaptohistidine (**35**), an intermediate in ovothiol A synthesis, and non-sulphur containing metabolites including valine (**36**) and pyroglutamic acid (**27**). Some of the proposed structures were supported by MSMS data, although fragmentation patterns could not be obtained for all metabolites due to low abundance (File S4). Figure 4 depicts proposed structures for some of the Bzn metabolites detected in 20 µM and/or 50 µM Bzn metabolomics analysis.

**Figure 5:**
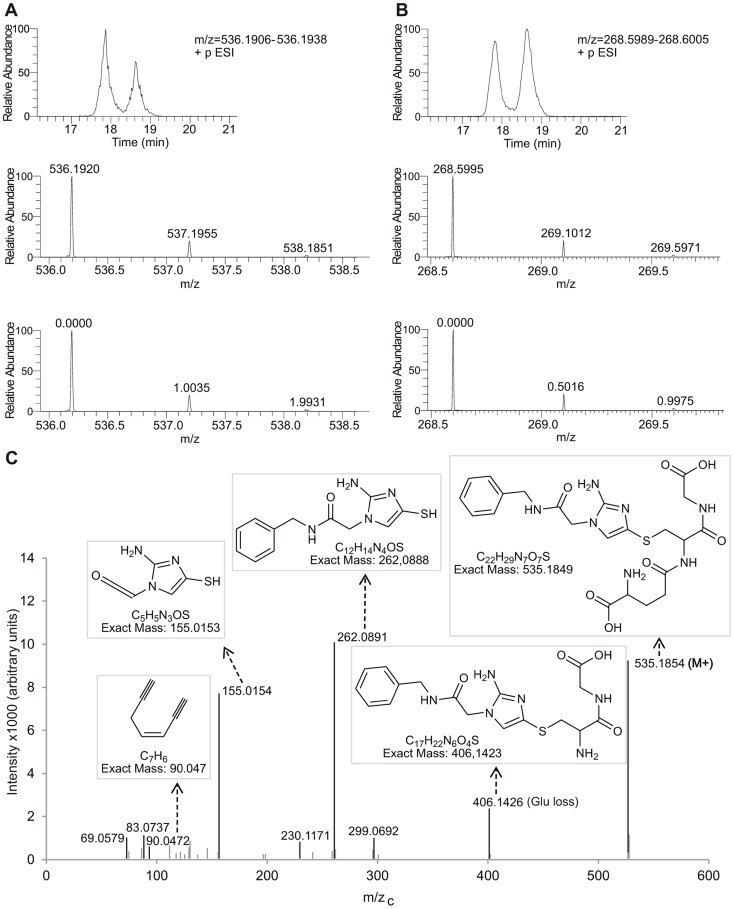
Identification of Bzn-glutathione adducts. A. Upper plot is a chromatogram obtained from a sample of Bzn treated parasites corresponding to m/z 536.1922 within a 3 ppm mass range (single-charged ion). Middle and bottom plots are magnified mass spectra in the regions of interest within RT 18.5–19. B. Chromatograms and m/z plots corresponding to m/z 268.5997 (di-charged ion) in a sample of Bzn treated parasites. In both A and B each ion is detected in two different but closely eluting chromatographic peaks with retention times centred at 17.8 and 18.6 minutes. Ionization charge states of the molecules are confirmed by the m/z difference for the isotopic ^13^C peaks, which are expected to be 1.0034 for mono charged ions and 0.5017 for di-charged ions (bottom plots). ^34^S isotopic peaks are also observed for both ions which are expected at 1.9959 m/z difference for mono-charged ions and 0.9979 for di-charged ions. C. MSMS fragmentation spectrum for precursor ion *m/z* 536.19. The m/z_c_ = *m/z*−1.007276 (proton mass). The proposed structures for the precursor ion (Bzn-glutathione adduct) and for some of the most intense fragments are shown. Exact theoretical mass and formula are included together with each fragment structure. A 4-C adduct is depicted though a 5-C adduct could be represented.

Many of the *in situ* detected Bzn-thiol conjugates display two different but close retention times, which likely correspond to structures equivalent to the ones described for reduced misonidazole adducts with glutathione [25]. These structures include the thiol moiety bound to the imidazole ring through either carbons at position 4 or position 5 (see Figure 4 for proposed structures). As an example, Bzn-glutathione adduct chromatograms, and MS and MSMS spectra are shown in Figure 5. Similar intensities were observed at the two different retention times where Bzn-glutathione adducts were detected, whereas Bzn-ovothiol A and Bzn-glutathionylspermidine adducts displayed very low intensity peaks at one of the retention times, which possibly results from preferred binding to one of the two imidazole carbons (4-C or 5-C).

## Discussion

Here we subject *Trypanosoma cruzi* to a HILIC chromatography coupled to high accuracy MS platform, which has been successfully used to analyse the cellular metabolomes of other trypanosomatids [34], [35], [36], [37]. This platform identified many different small molecules, including low abundance metabolites, and allowed their quantitative comparison. Medium composition [38], extraction method [21] and the analytical platform used for analysis [39] all influence output, and hence a standardized protocol for trypanosomatid metabolomics was applied. To minimize changes due to sample handling (that can provoke cell leakage and metabolite draining), parasite pellets were not washed before metabolite extraction, and hence medium samples were always included as controls.

Amino acids and their derivatives (peptides, thiols and polyamines) were the most abundant class identified, representing 56.7% of all detected metabolites. The high rate of endocytosis and proteolytic activity [40], combined with high protein and peptide content of culture medium probably explains this abundance. We also identified and semi-quantitatively analysed nearly one thousand compounds, including a wide array of hydrophobic and hydrophilic molecules including many lipids, carbohydrates and nucleotides among others. In spite of the HILIC based platform used here being sub-optimal for very lipophilic or highly acidic or poly-phosphorylated molecules, it is clearly a relatively straight forward means for the systematic analysis of the metabolic state of *Trypanosoma cruzi* suitable for studying many aspects of the biochemistry and pharmacology of these parasites. Improvements in chromatography, such as the introduction of pHILIC columns that can be operated at high pH and thus improve resolution of phosphorylated compounds and carboxylic acids could further improve coverage of the metabolome [39].

Benznidazole is the drug of choice for treatment of both acute and chronic Chagas disease. It has been widely agreed that Bzn exerts activity through induction of reductive stress, involving covalent modification of cellular macromolecules by intracellular reduced metabolites of the parent compound [12], [13]. Bzn can arrest protein, RNA and DNA synthesis, as well as promote damage to both nuclear and kinetoplast DNA and macromolecule degradation [41], [42], [43], [44]. Here we used 20 µM and 50 µM over a 6 h incubation period with parasites to detect induced changes. A recent report shows that DNA damage, including double strand breaks, was found in *T. cruzi* after treatment with 240 µM Bzn for up to 72 h [44]. 8-oxoguanine was encountered in *T. cruzi* DNA after Bzn exposure and augmented levels of oxidized deoxyguanosine triphosphate (8-oxodGTP) are proposed as the main cause for the observed DNA damage. Here we failed to identify key oxidation products of cellular nucleotides, although using lower drug concentrations for lower exposure times might be responsible for this difference.

A type I nitroreductase (NTRI) has been shown to play a major role in the cytotoxicity of both Bzn and Nfx, and diminished NTRI activity correlates with nitroheterocycle cross resistance [14], [15]. Hall and Wilkinson (2012) studied the products of Bzn reduction catalysed by *T. brucei* and *T. cruzi* NTRI in a cell-free system. Two consecutive two-electron reductions proceed with no oxygen consumption, and lead to the formation of a hydroxylamine derivative that then rearranges to form a dihydroxy-dihydro derivative. This metabolite can decompose to give glyoxal, a well-known toxic metabolite, postulated to contribute to the pleiotropic effects that Bzn induces in trypanosomes [17]. However, the biotransformation of Bzn in *T. cruzi* has not been directly investigated, and was addressed in this metabolomic study.

If Bzn-related glyoxal damage occurs in *T. cruzi*, covalent conjugates of glyoxal with small cellular molecules would be expected to arise after Bzn treatment. However, we could not detect signals belonging to the most common products of glyoxal conjugation with any measured cellular metabolites including nucleotides, nitrogenated bases or amino acids. The raw data was scanned for low intensity signals but also failed to detect any candidate glyoxal adducts. Deoxy-guanosine or guanosine adducts, the major adducts of glyoxal and methyl-glyoxal *in vivo* [45] were not found. Likewise, carboxy-methyl-cysteine, which is considered a marker for glyoxal cell damage in mammals [46], was not detected. Possible roles for the trypanothione-dependent glyoxalase system operative in *T. cruzi* [47] in detoxifying glyoxal can be considered, although trypanothione-glyoxal conjugates were not evident in the metabolomics data. It remains possible that glyoxal conjugation to cellular macromolecules, not detected in the small molecule population we measure, is associated with activity. An ion corresponding to N-benzyl-2-guanidinoacetamide (**5**) (m/z 206.12 RT 11.7 min) was detected. This molecule is expected to be formed as a consequence of glyoxal release from the dihydroxy-dihydro derivative of Bzn. However, the relative intensity of this ion (**5**) was an order of magnitude lower than the dihydroxy-dihydro derivative (**3**), and 100-fold lower than the reduced amine derivative (**1**). Whilst absolute quantification of Bzn metabolites was not performed (due to a lack of authentic standards), this low abundance strongly suggests that glyoxal production is not the major route of Bzn metabolism in *T. cruzi*. The equilibrium transfer of glyoxal from reduced Bzn reported in both *in vitro* analyses was slow and inefficient [17], [32]. Also, the covalent interactions of Bzn with lipids, nucleic acids and proteins that have been reported up to now were measured using 2 -^14^C labeled Bzn [12] and thus do not include possible adducts arising from glyoxal conjugation as this involves only carbons at position 4 and 5 of the imidazole ring. Altogether, if glyoxal molecules are released *in vivo* after Bzn reduction by *T. cruzi*, their fate remains uncertain.

Our metabolomics analysis revealed that Bzn stimulated relatively few changes to metabolite concentrations within *T. cruzi* in the conditions tested. These included diminished abundance of the low molecular weight thiols trypanothione, homotrypanothione and cysteine. Although these metabolic changes may result from inhibition of enzymes involved in the biosynthesis of these thiols; it is more likely that the perturbations are due to direct thiol depletion by adduct formation. The major class of metabolites to increase in abundance were glutamyl dipeptides, suggesting upregulation of the γ-glutamyl cycle involved in glutathione recycling, which may result from turnover of Bzn-glutathione and Bzn-trypanothione adducts. These results indicate that the major metabolic perturbation of Bzn was detected in the glutathione (and trypanothione) pathway. Additional metabolic perturbations were observed following high-dose Bzn treatment, including accumulation of long-chain acyl-carnitines and depletion of some sterols. The role of these lipid perturbations in the mechanism of Bzn activity is not known, although it is noted that lipid, and especially sterol metabolism is already a validated drug target for *T. cruzi* [48]. Interestingly, the ergosterol biosynthesis inhibitor, posaconazole, exhibited synergistic activity when administered with Bzn in a *T. cruzi* mouse model [49], potentially supporting a link between Bzn action and sterol metabolism.The putatively identified acyl-carnitines have not been previously reported in *T. cruzi* and further investigation of the novel trypanosomatid biochemistry discovered here may provide new opportunities for drug target discovery for Chagas disease.

In addition to endogenous metabolites, a high number of novel molecules, all drug derived metabolites, were detected in parasites treated with Bzn. We were able to assign structures to some of these molecules. Many represented reduced Bzn derivatives previously detected using *in vitro* systems. For instance, a molecule with a mass of 264 Da previously assigned to a dihydroxy-dihydro derivative was detected (**3**). This molecule, 2-(2-amino-4,5-dihydroxy-4,5-dihydroimidazol-1-yl)-N-benzylacetamide, was first described as the main product of Bzn following chemical, radiochemical or electrochemical reduction [32]. The nitroreductase NTRI reduces Bzn *in vitro* in the presence of NADH to a number of products, including the dihydroxy-dihydro derivative as the major product [17]. The MSMS fragmentation pattern of the ion obtained from our *T. cruzi* samples (m/z 264.12, RT 13 min) (**3**) is equivalent to the fragmentation pattern obtained for the *in vitro* product of trypanosomal NTRI. Other metabolites obtained after Bzn NTRI catalyzed reduction are the hydroxy and hydroxylamine derivatives (**11**, **19**), N-benzyl-2-guanidinoacetamide (**5**) and 2-amino-N-benzylacetamide (**22**), which may arise after reductive fragmentation as reported with misonidazole [50]. Ions corresponding to all of these molecules were detected in our Bzn-treated parasites. High intensities of these metabolites in Bzn treated parasites supports the hypothesis of a major contribution of TcNTRI in the reduction and toxicity of Bzn towards *T. cruzi*.

Covalent adducts of reduced Bzn metabolites with the principal low molecular weight thiols were prominent. Cysteine, glutathione, γ-glutamylcysteine, glutathionylspermidine, trypanothione and Ovothiol A adducts were all detected, consistent with the loss of free-thiols following Bzn and Nfx treatment of different stages of *T. cruzi* [51], [52], [53]. Buthionine sulfoximine (BSO), an inhibitor of glutathione synthesis, showed synergistic effects upon co-administration with Nfx in a murine model [54] and a correlation of the intracellular thiol concentration in different strains and the toxicity of these compounds was also suggested [51]. Here, we demonstrate the intracellular generation of Bzn-thiol conjugates, and the accompanying depletion of endogenous thiols, as major factors associated with drug action in *T. cruzi* (Figures 4 and 5).

The relative roles of enzymatic (e.g. through glutathione and trypanothione-S-transferases) and non-enzymatic production of these thiol conjugates is not certain, but non-enzymatic conjugation to macromolecular thiols, e.g., cysteine in proteins, is also likely to contribute to the mode of action of the drug as has been reported for metronidazole and other nitroimidazoles [55], [56]. Low molecular weight cysteine-rich proteins in *T. cruzi* were shown to have these residues blocked when exposed to Nfx or Bzn, again indicating binding of metabolites from these drugs to macromolecular cysteine residues [57]. Many enzymes rely on active cysteine residues to exert their functions including the tryparedoxin peroxidase-tryparedoxin system, among others, and could be targeted by Bzn metabolites [58]. Interestingly, however, our metabolite profiling of treated cells indicated relatively little change to cellular metabolism which suggests that most cellular enzymes have not been affected sufficiently to change the flux through most metabolic pathways. Also, some of the observed Bzn-derived metabolites included covalent adducts with small metabolites other than thiols including pyroglutamic acid adducts and possibly valine adducts. This pleiotropic reactivity of Bzn derived metabolites could also account for some of its toxicity and for the binding of Bzn to other macromolecules including lipids and nucleic acids.

In addition to the Bzn-derivatives previously noted as NTRI products, other metabolites were also found in our analysis. Among these, 2-(2-amino-1H-imidazol-1-yl)-N-benzylacetamide (**1**) has one of the most intense signals detected. This molecule arises after a six electron reduction of Bzn and was only detected in a low yield after zinc chemical reduction of Bzn, and was not detected after radiation or electrochemical reduction [32]. The molecule was, however, detected after Bzn metabolism by mammalian tissues, for which NADPH–cytochrome P450 reductases and cytochrome P450 may be responsible. As a stable molecule it is proposed to be non-toxic [59]. Interestingly, overexpression of a NADPH–cytochrome P450 reductase (TcCPR-B) in *T. cruzi* epimastigotes decreased sensitivity to Nfx and Bzn [60]. A role of TcCPR-B in the detoxification of Bzn was therefore suggested but the mechanism remains unclear, and reduction to an amine non-toxic product could explain the observed phenotype. The methoxy derivatives of the dihydroxy-dihydro form of Bzn may also represent detoxification products. Other enzymes might also have roles in the generation of the reduced Bzn metabolites, such as the two structurally related enzymes TcOYE [61], [62] and TcAKR, which binds *in vitro* to immobilized Bzn (Trochine A., Faral-Tello P. and Robello C. unpublished data). As *T. cruzi* shows differences in pathogenicity, virulence, transmissibility and drug sensitivity through its six major genotypic divergent lineages [63], the complex pattern of Bzn metabolism that we have unravelled here could help explain the significant variability in sensitivity to this drug in the different parasite-isolates across Latin America [64], [65]. Investigation of the enzymes responsible for the formation of each of the Bzn metabolites and its possible implications on Bzn toxicity or resistance should be straightforward using the metabolomics platform and data obtained in this work, together with overexpressing or knock-out parasite lines.

### Conclusions

Metabolomics technology has allowed us to gain further insight into Bzn mechanism of action. The MS based metabolomics analysis is a powerful tool and can be used to analyse the mode of action of different types of drugs in *Trypanosoma cruzi*. These in turn should help us understand resistance mechanisms as well as the natural variation in *T. cruzi* susceptibility to nitroheterocycles and to improve available and future drugs. Through this work we have shown that Bzn is extensively metabolized to a number of molecules once it enters *T. cruzi*. These molecules include reduction products and covalent adducts with low molecular weight thiols and other small molecules. In addition, the metabolomics analysis of endogenous metabolites identified low molecular weight thiol depletion and turnover as the major metabolic impact of Bzn treatment. We here propose that the covalent binding of Bzn with low molecular weight thiols as well as with protein thiols is a primary cause of the drug's toxicity against *T. cruzi*.

## Supporting Information

Figure S1 **Principal Components Analysis plot.** PCA Score Plots were generated with normalized MS peak intensity data using Metaboanalyst [68], [69]. PCA is an unsupervised clustering or classification method which projects complex high-dimensional data to a new coordinate system with fewer dimensions. The projection direction is calculated to maximize the data variance in just the first few dimensions, called principal components (PC). Scores represent the original data in the new coordinate system and are weighted average of the original variables. Samples: Med: fresh medium, cBc: control of non-treated parasites (20 µM Bzn added before metabolite extraction), cBt: 20 µM Bzn treated parasites, cTc: control of non-treated parasites, mBc: cBc spent medium, mBt: cBt spent medium.
(TIF)

File S1 **Ideom file showing all metabolites identified in Bzn 20 µM treatment of** ***T. cruzi*** **.**
(XLSB)

File S2 **Ideom file showing all metabolites identified in Bzn 50 µM treatment of** ***T. cruzi*** **.**
(XLSB)

File S3 **File showing data from Bzn metabolites collected from raw files.**
(XLSX)

Table S1 **Samples.**
(PDF)

File S4 **File showing all collected analysed MSMS data.**
(XLSX)
