# Supplementary figures and images for: Benznidazole Biotransformation and Multiple Targets in Trypanosoma cruzi Revealed by Metabolomics
Source: PLoS Negl Trop Dis. 2014 May 22;8(5):e2844. doi: 10.1371/journal.pntd.0002844 (PMC4031082; doi:10.1371/journal.pntd.0002844)

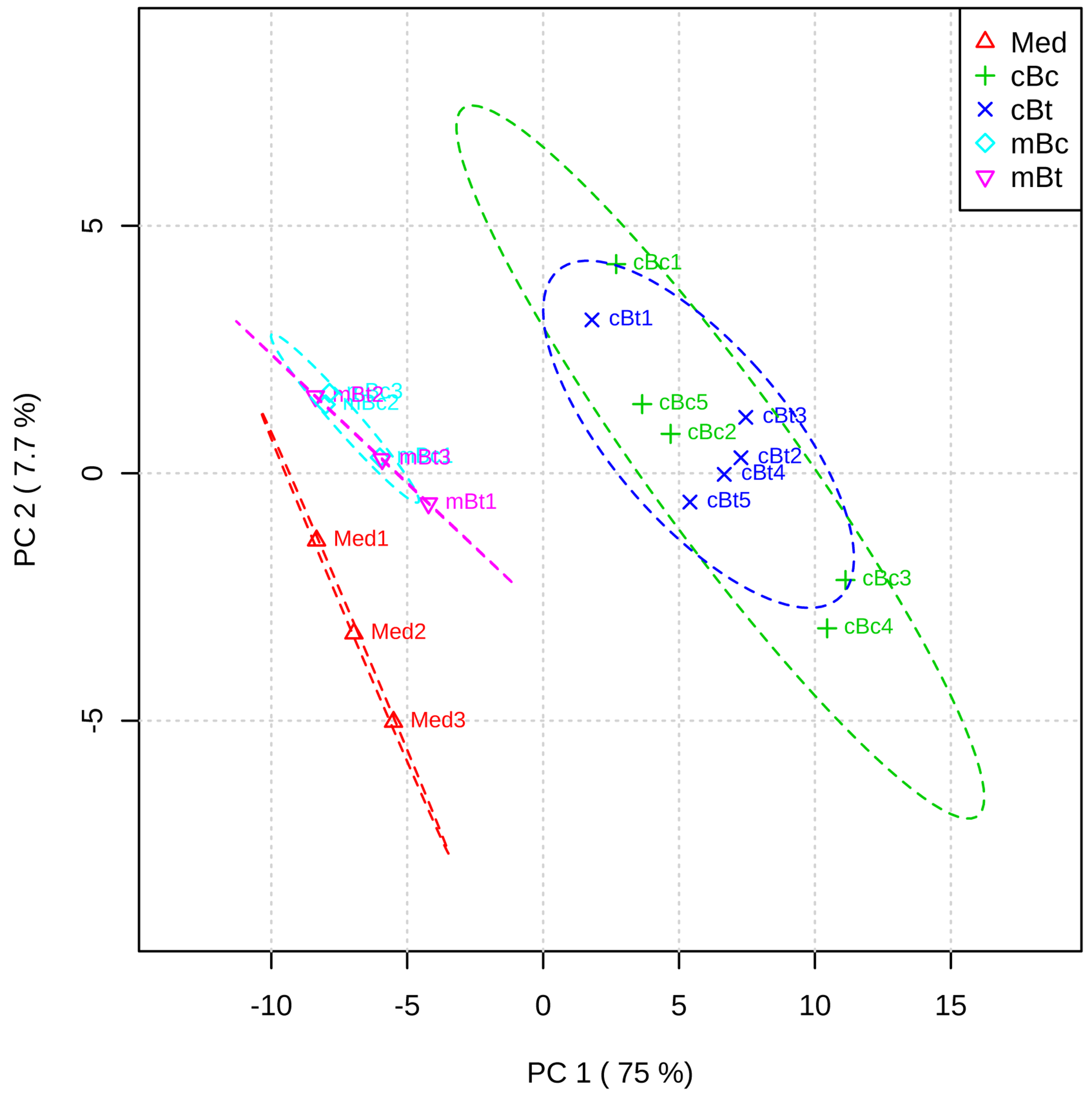

Supplement: Figure S1 — Principal Components Analysis plot. PCA Score Plots were generated with normalized MS peak intensity data using Metaboanalyst [68], [69]. PCA is an unsupervised clustering or classification method which projects complex high-dimensional data to a new coordinate system with fewer dimensions. The projection direction is calculated to maximize the data variance in just the first few dimensions, called principal components (PC). Scores represent the original data in the new coordinate system and are weighted average of the original variables. Samples: Med: fresh medium, cBc: control of non-treated parasites (20 µM Bzn added before metabolite extraction), cBt: 20 µM Bzn treated parasites, cTc: control of non-treated parasites, mBc: cBc spent medium, mBt: cBt spent medium. (TIF) [file pntd.0002844.s001.tif]
