# Supplementary material for: Benznidazole Biotransformation and Multiple Targets in Trypanosoma cruzi Revealed by Metabolomics
Source: PLoS Negl Trop Dis. 2014 May 22;8(5):e2844. doi: 10.1371/journal.pntd.0002844 (PMC4031082; doi:10.1371/journal.pntd.0002844)
Supplement: Table S1 — Samples. (PDF) [file pntd.0002844.s005.pdf]

Table S1. Samples.

Sample name	Description
cBt	Treated parasites (20 µM Bzn)
cBc	Non-treated parasites with Bzn added after metabolic quenching (20 µM Bzn)
cTc	Non-treated parasites with an unrelated drug added after metabolic quenching
mBc	Spent medium from cBc samples
mBt	Spent medium from cBt samples
Med	Fresh medium
Sol	Extraction solvent
QC	Quality control, pool of all cell samples
cBzt	Treated parasites (50 µM Bzn)
cBzc	Non-treated parasites with Bzn added after metabolic quenching (50 µM Bzn)
cBec	Non-treated parasites with an unrelated drug added after metabolic quenching
